# The Relevance of Dual Diagnoses among Drug-Dependent Patients with Sleep Disorders

**DOI:** 10.3390/jcm9092862

**Published:** 2020-09-04

**Authors:** Carlos Roncero, Llanyra García-Ullán, Alberto Bullón, Diego Remón-Gallo, Begoña Vicente-Hernández, Ana Álvarez, Amaya Caldero, Andrea Flores, Lourdes Aguilar

**Affiliations:** 1 Psychiatry Service, University of Salamanca Health Care Complex, Paseo de San Vicente 58-182, 37007 Salamanca, Spain; mlullan@saludcastillayleon.es (L.G.-U.); abullons@saludcastillayleon.es (A.B.); bvicenteh@saludcastillayleon.es (B.V.-H.); aialvarez@saludcastillayleon.es (A.Á.); maguilar@saludcastillayleon.es (L.A.); 2 Psychiatry Unit, School of Medicine, University of Salamanca, Campus Miguel de Unamuno C/ Alfonso X El Sabio s/n, 37007 Salamanca, Spain; afc@usal.es; 3 Institute of Biomedicine, University of Salamanca, Paseo de San Vicente, 58-182, 37007 Salamanca, Spain; diego_biscab@hotmail.com (D.R.-G.); amayacaldero@hotmail.com (A.C.); 4 Psychiatry Service, Zamora University Health Care Complex, Hernán Cortés Street, 40, 49071 Zamora, Spain

**Keywords:** dual disorders (DD), insomnia, sleep disorders (SD), benzodiazepine use disorder (BUD)

## Abstract

Background: Sleep disorders are often associated with drug use. Nearly 70% of patients admitted for detoxification report sleep problems. Dual disorder (DD) is the comorbidity between mental disorders in general and disorders related to psychoactive substance use. The association between substance use and sleep disorders (SD) appears to be bidirectional. Our objective is to analyze the association between sleep disturbance history and drug use pattern (alcohol, cannabis, opioids, and cocaine). Methods: Analysis of data in the first interview at the Addictions Unit of the Department of Psychiatry at the University of Salamanca Health Care Complex between October 2017 and January 2020. The sample consists of 398 patients. We studied the association between different variables: origin of patients (Inpatient Dual Diagnosis Detoxification Unit (IDDDU) vs. Outpatient Drug Clinic (ODC), presence of affective disorder, psychotic disorder, type of drug used, and treatment. Results: Of patients with DD, 62% had more delayed sleep induction, sleep fragmentation, early awakening, and nightmares. Outpatients had more difficulty falling asleep because, in many cases, they had not previously sought any medical assistance. On the other hand, 67% of the patients with insomnia presented depression. Conclusions: There is evidence of a harmful association between DD and SD.

## 1. Introduction

Sleep disorders are associated with drug use. Almost 70% of all the patients who are admitted for detoxification have sleep disorders [1,2]. The association between the use of substances and insomnia (here used as sleep disorders in general) seems to be bidirectional [3], since sleep disorders increase the risk of developing substance use disorders [4], and the use of substances causes sleep disorders [5]. Long-term abstinence may reverse some sleep disorders [6]. On the other hand, drugs are known to be used as self-medication to relieve some sleep disorders [7]. Sleep disorders may also be a risk factor for a relapse in substance abuse [8]. Insomnia, and particularly delayed sleep induction (DSI), is related to a relapse in alcohol use [9]. It has also been associated with relapses in the use of cocaine [10], and there is evidence showing that improvements in sleep disorders may predict abstinence in opioid-dependent patients [11].

Insomnia is present in several stages of alcohol use [12]. In turn, alcohol is used by 45% of patients with substance use disorders, as self-medication for their sleep disorders [8]. As the alcohol consumption becomes chronic, it decreases its hypnotic effect. The rates of insomnia among alcoholics range between 35 and 70% [13]. These rates are higher than those observed for the general population (15–30%) [13]. Patients report difficulty falling asleep, sleep fragmentation, daytime sleepiness, bad quality of sleep and, sometimes, hypersomnia [14]. Knowing the changes in circadian rhythms caused by alcohol can help us in its treatment. After a single acute intake of alcohol, changes in biological rhythms are reflected in melatonin and cortisol secretions and central body temperature (CBT) rhythms. These alterations are more severe during alcohol use disorder (AUD) and persist over time. Opposite patterns of the physiological relationship of melatonin between daytime and night-time discharge have been observed (N/D < 1 ratio). Resynchronization of circadian cortisol and CBT rhythms occurs approximately one month after leaving alcohol. Disruption of circadian melatonin rhythms may persist for 3–12 weeks [15].

Sleeping problems associated with alcohol use disorder are some of the most refractory disorders [9]. Cognitive behavioral therapy for insomnia (CBT-I) has been described as the first line treatment. On the other hand, mirtazapine, gabapentin, and quetiapine have a moderate level of evidence. Benzodiazepines should be avoided [16].

As in the case of alcohol, the use of cannabis improves insomnia, particularly when used over a short period of time [17]. However, the chronic consumption of cannabis is associated with negative effects on sleep that are more visible during abstinence. These effects are present during the interruption of cannabis use, particularly in habitual cannabis users, but also in people exposed to low doses [18,19].

Cocaine abstinence is behind many complaints related to sleep. During the first week of abstinence, patients may show insomnia, nightmares and, sometimes, hypersomnia. They also report depressive symptoms, fatigue, increased appetite, and agitation episodes [20]. Eighty percent of the people with an increased need for sleep during cocaine abstinence in the early stages self-medicate with alcohol and opioids [21]. When the patients remain abstinent, the quality of sleep improves [22], and sleeping routines return to normal after several weeks [21].

There are a limited number of studies on the effects of abstinence and chronic use of opioids. Asaad et al. described alterations including insomnia, hypersomnia, increased latency, and decreased duration of sleep after three weeks of abstinence [23]. The quality of sleep was studied in patients 5 days after starting treatment with methadone. Patients without previous sleep disorders obtained lower scores in the Pittsburgh Sleep Quality Index (PSQI) and showed daytime sleepiness in the Epworth Sleepiness Scale (ESS) [24].

On the other hand, during the first stages of methadone detoxification [25] patients reported inadequate quality and quantity of sleep, as well as difficulties falling asleep [26]. After long periods of treatment with methadone, it was observed that this difficulty falling asleep lasted from 6 to 12 months [27].

Among the anti-depressants that can cause insomnia are those that inhibit the reuptake of serotonin and noradrenaline (SNRIs), noradrenaline reuptake inhibitors (NRIs), monoamine oxidase inhibitors (MAOIs), selective serotonin reuptake inhibitors (SSRIs), and tricyclic antidepressant activators (TCAs). In contrast, antihistamine-active antidepressants, such as the sedative tricyclic antidepressants, mirtazapine, mianserin, and serotonin 5-HT2 receptor antagonists, such as trazodone and nefazodone, rapidly improve sleep. Some patients already show an improvement in sleep quality after the first dose of the drug [28], which was observed with mirtazapine in relation to the faster onset of antidepressant action [29].

On the other hand, antidepressants can cause sleep disorders or worsen existing ones. Mianserin and mirtazapine can induce restless leg syndrome in up to 28% of patients. It has also been described for SSRIs as well as venlafaxine [30]. SSRIs, SNRIs, and ACTs induce or exacerbate sleep bruxism and alter the regulation of muscle tone during REM sleep [31,32]. In addition, although antidepressants are recommended for the treatment of post-traumatic sleep disorder, they can induce nightmares, especially with mirtazapine.

The relationship between insomnia, psychiatric disorders (mainly depression and anxiety), and drug use disorder has already been described [33,34,35]. Winkour et al. observed that 100% of the patients in a sample of 1257 people with depression also presented comorbid insomnia [36]. The relationship between sleep disorders and psychiatric disorders is gaining increased attention, particularly as evidence shows that insomnia is not just a typical symptom of depression or other psychiatric disorders, but that it may actually be a predictive factor (or an independent risk factor) for the development of other psychiatric disorders, including substance use [37].

Our objective is to analyze whether patients with dual disorder present more sleep alterations than non-dual addicts, and to assess the types of sleep disorders (DSI, sleep fragmentation, early awakening, and nightmares) depending on the accompanying disorder, consumed substance, or both. We will also study the presence of sleep disorders based on whether the patients are receiving outpatient or inpatient care.

## 2. Materials and Methods

The study included patients diagnosed with substance use disorder based on the Diagnostic and Statistical Manual of Mental Disorders (DSM)-criteria who visited the Outpatient Drug Clinic (ODC) or were admitted into the Inpatient Dual Diagnoses Detoxification Unit (IDDDU) of the Salamanca Health Care University Complex from October 2017 to January 2020. Dual disorder (DD) is the comorbidity between mental disorders in general and disorders related to psychoactive substance use [38]. Patients who requested voluntary discharge on the first day of admission were excluded from the study, as well as those who had difficulties answering the questions due to cognitive or language alterations, and those who only cooperated partially when being assessed.

**Figure 1:**
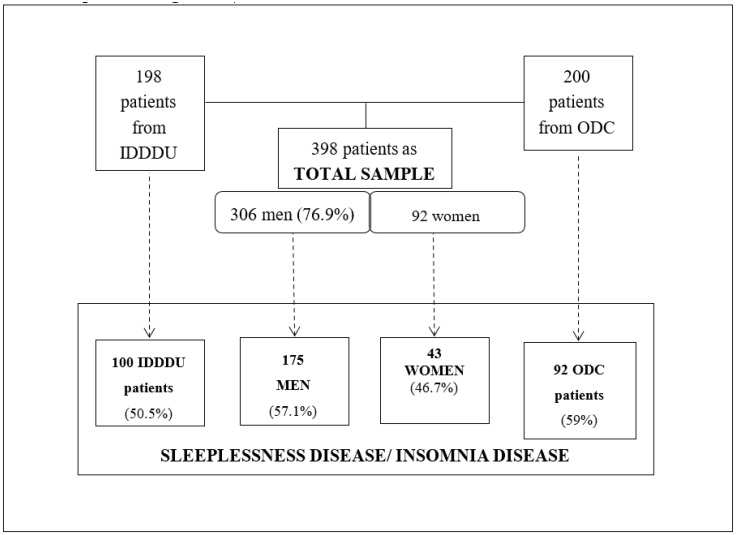
Flowchart of gender and origin characteristics from the sample. Abbreviations; IDDDU: Inpatient Dual Diagnoses Detoxification Unit; ODC: Outpatient Drug Clinic.

The research was approved by the Ethical Committee of Salamanca University Health Care Complex according to the Declaration of Helsinki (2075/A/19). Figure 1 shows gender and the origin (ODC/IDDU) of the study sample.

The assessment included a structured interview with 16 items, some of which had yes/no answers: presence of a dual disorder being treated in a program; presence of non-addictive mental disorder; affective disorder; psychotic disorder; patient from the ODC; patient from the IDDDU; type of addiction (alcohol, cannabis, cocaine, and heroin); alcohol withdrawal score ≥ 10 in the CIWA-AR scale (Revised Clinical Institute Withdrawal Assessment for Alcohol Scale) [39]; cannabis withdrawal syndrome; delayed sleep induction; sleep fragmentation; early awakening; and nightmares. Other items showed multiple options: occupational status (working, unemployed, on leave, or retired); treatment in the first interview; age; amount of cannabis consumed; amount of alcohol consumed; amount of cocaine consumed; amount of opioids consumed; and amount of benzodiazepines consumed. Multiple drug-users are codified when abuse or dependence on more than one drug exists (not including tobacco).

The diagnosis of insomnia is made when the patient reports dissatisfaction with sleep (difficultly to sleep or to remain asleep, when the sleep was fragmented, when there was an early awakening or nightmares) and other daytime symptoms (e.g., fatigue, decreased energy, mood disturbances and reduced cognitive functions such as impaired attention, concentration, and memory) for at least 3 nights per week and that lasts for more than 3 months [40]; all the data were collected and analyzed with SPSS version 25. The comparative analysis was carried out with the nonparametric chi-squared test.

Binomial logistic regression was used, including the variables that were significantly associated with insomnia: origin of the patient (ODC/IDDDU), presence of dual disorders; previous treatment in a different center; occupational status, pharmacological treatment in the first interview, and amount of benzodiazepines consumed. 

## 3. Results

The sample included 398 patients (76.9% men; mean age: 47 years). Of these, 198 patients (49.7%) came from the IDDDU, and 200 (50.3%) came from the ODC. Men presented more sleep disorders, represented in the column labeled “Insomnia/Any sleep disorder” (57.1%), than women (46.7%) (Table 1). The highest rate of Insomnia/Any Sleep Disorder was observed in the 48–57 age group (35.8%). Patients who consumed 1–5 units of cannabis had the most sleep disorders, particularly DSI and sleep fragmentation (Table 2). The same was true for patients with alcohol use disorder: those who consumed up to 20 alcohol units per day presented more sleep disorders than those who consumed 41 alcohol units per day or more. That is, lower consumption rates were correlated with higher rates of sleep disorder.

Delayed sleep induction (*χ*^2^ = 10.48; *p* = 0.01), early awakening (*χ*^2^ = 5.598; *p* = 0.018), and nightmares (*χ*^2^ = 3.898; *p* = 0.048) were more common in outpatients (Table 3). In our sample, 61.6% of the patients had dual disorders and presented more sleep disorders than non-dual patients: insomnia (*χ*^2^ = 4.267; *p* = 0.039), difficulty falling asleep (*χ*^2^ = 2.877; *p* = 0.09), sleep fragmentation (*χ*^2^ = 4.862; *p* = 0.027), early awakening (*χ*^2^ = 7.554; *p* =0.006), and nightmares (*χ*^2^ = 12.988; *p* = 0.000).

With regard to cocaine use, those who consumed 1 g per week showed higher sleep disorder rates (12.3% of nightmares group) than those that consumed more cocaine. Opioid consumers represent between 21.2 and 26.7% of sleep disorders groups, particularly conciliation insomnia.

Similarly, patients with a dual disorder who had been treated in a different program (N = 172) showed higher rates of delayed sleep induction (*χ*^2^ = 9.291; *p* = 0.02), sleep fragmentation (*χ*^2^ = 4.180; *p* = 0.041), early awakening (*χ*^2^ = 9.913; *p* = 0.02), and nightmares (*χ*^2^ = 10.814; *p* = 0.001) than non-dual patients.

The most commonly used drug for insomnia in the initial interview was alprazolam (4.3%), followed by diazepam (3.3%). The use of benzodiazepines was clearly associated with all types of insomnia (*χ*^2^ = 9.848; *p* = 0.043), delayed sleep induction (*χ*^2^ = 15.21; *p* = 0.04), sleep fragmentation (*χ*^2^ = 16.924; *p* = 0.002), early awakening (*χ*^2^ = 13.316; *p* = 0.010), and nightmares (*χ*^2^ = 18.980; *p* = 0.001). Consuming a larger quantity of benzodiazepines was associated with all types of sleep disorder.

The presence of nightmares during sleep is significantly associated with an ambulatory treatment (*χ*^2^ = 3.898; *p* = 0.0048), being a woman (*χ*^2^ = 7.069; *p* = 0.008), having a dual disorder (*χ*^2^ = 10.814; *p* = 0.001), being treated with benzodiazepines (*χ*^2^ = 18.980; *p* = 0.001), and alcohol abstinence (*χ*^2^ = 6.488; *p* = 0.011).

Finally, multivariate analysis is depicted in Table 4. Benzodiazepine use disorder (*p* = 0.029; OR = 0.354), treatment with trazodone (*p* = 0.031; OR = 0.129), and treatment with pregabalin (*p* = 0.031; OR = 0.129) were protector factors for insomnia. Having a concomitant psychiatric disorder (*p* = 0.039; OR = 1.553) and the origin of the patients were risk factors for some sleep disorders, such as conciliation insomnia (*p* = 0.01; OR = 1.991), early awakening (*p* = 0.019; OR = 1.80), and nightmares (*p* = 0.050; OR = 1.656).

## 4. Discussion

In our sample, 62% of the patients had dual disorders and presented more sleep disorders than non-dual patients: insomnia, difficulty falling asleep, sleep fragmentation, early awakening, and nightmares. Occupational status and consuming larger amounts of benzodiazepines were associated with the presence of sleep disorders; while being a woman, symptoms of alcohol withdrawal, and personality disorders were associated with the presence of nightmares. On the other hand, 67.3% of the patients who reported insomnia had a dual disorder, and this rate is much higher than that of the general population (9–12%) [34,41].

The most common disorders in the dual population were affective and psychotic disorders, which is in line with what was reported in previous studies [2,35,42,43]. Approximately 20% of all addict patients with sleep disorders present some symptom of depression [44,45,46,47]. Staner et al. already observed that having a psychiatric disorder, and particularly depression, was the most important risk factor for insomnia [47]. Therefore, it may be said that the relationship between insomnia and depression in addict patients is bidirectional.

With regard to personality disorders, these were associated with sleep fragmentation and, more notably, nightmares. This last finding coincides with what has been observed in other studies that described a higher number of admissions of addict patients with sleep disorders and a comorbid personality disorder [2].

The presence of nightmares was also associated with being a woman. This finding had already been observed in women with alcohol and other drug disorders. They did not only report having nightmares, but had in many cases been diagnosed with depression, personality disorders, and psychosis [48]. It is important to highlight that the main type of drug that was used had an influence on the results. In this regard, in patients with alcohol use disorders, the most common alterations were sleep fragmentation and delayed sleep induction (62%). In the same vein, in most of the previous studies, the prevalence of insomnia in patients with an alcohol use disorder ranged between 35 and 75% [12], because the patients often used alcohol as self-medication to sleep [7,49,50]. Patients with a cannabis use disorder presented lower rates of insomnia (29.8%) than those who did not consume it (70.2%), although the chronic use of cannabis is known to be associated with negative effects on sleep, particularly during periods of abstinence. Cannabis may improve subjective complaints about sleep when used over short periods of time [51,52]. Nightmares are generally the most common sleep disorders during abstinence [53]. They usually start 1–3 days after stopping consumption [53,54,55], they reach their peak after 2–6 days, and they last for 4–14 days [53]. Other studies report that difficulties in falling asleep last around 43 days [56] and nightmares may last up to 45 days [53]. As a consequence, there are generally relapses in the use of alcohol and cannabis to fall asleep [56].

In patients with cocaine use disorder, delayed sleep induction was the most common disorder, with the highest rates among patients who consumed over 6 g/week (11.1%). Eighty percent of the patients who wanted to sleep more during a period of abstinence from cocaine self-medicated in the early stages with alcohol and opioids [57,58,59]. No differences were observed regarding the type of insomnia in patients with opioid use disorder. Although there is little evidence on this topic, an association has been described between heroin use and sleep disorders [60,61,62], particularly falling asleep and maintaining sleep during the first stages of detoxification with methadone [62].

Some authors have described that multiple admissions to detoxification units are associated with more sleep disorders, with promotes substance use and relapses [1,2] and leads to a worse evolution of the addiction [2]. However, in our case, outpatients reported more difficulties falling asleep, and the sleep was of poorer quality than in hospitalized patients.

Benzodiazepines are the most widely sold group of drugs for sleep disorders, as has already been described in other series of addict patients [2] and mental health patients [63]. Benzodiazepine use disorder is common in patients who are being treated for a different addiction. It is associated with complications such as overdoses and suicide attempts. Although these drugs may modulate sleep disorders, they are not recommended for the treatment of insomnia in addict patients [64,65,66], and the risk and possibilities of their misuse must be taken into consideration [1].

This study must be analyzed considering its limitations, since it is a cross-sectional analysis that does not make it possible to establish causal associations regarding the influence of drug abstinence or the psychopathological evolution of the patients. We may highlight that the assessment of the presence of insomnia did not include electrophysiological tests such as polysomnography and actigraphy. However, electrophysiological tests are recommended as a second choice, because the diagnosis of insomnia is mainly clinical, and it is based on the history of the patient.

Nevertheless, this study has some strengths, since it includes a large sample of unselected real-world outpatients and inpatients who required treatment. In this regard, it is important to highlight that there are very few analyses that describe the prevalence of insomnia in dual patients and that specify the type of sleep disorder [67]. Most of the studies published in the literature only include alcohol-dependent patients.

## 5. Conclusions

Patients with a dual disorder present sleep disorders (delayed sleep induction, sleep fragmentation, early awakening, and nightmares) with a significantly higher rate than non-dual patients. On the other hand, 67.3% of the patients who reported insomnia had an associated psychiatric disorder, mostly affective disorders, with psychopathology as the most common one. 

The main drug associated with sleep disorders was alcohol (65.1%), and nightmares showed a significant association with alcohol withdrawal syndrome.

An association was found between sleep disorders and the origin of the patients, and outpatients showed more difficulties falling asleep.

The presence of a dual disorder is relevant for the appearance of sleep disorders in addict patients and vice versa. In patients with sleep disorders and substance use disorders, the existence of a dual pathology must be taken into account.

Benzodiazepine use disorder was significantly associated with all the types of sleep disorders. An association was also observed between insomnia and the use of trazodone and pregabalin. 

Sleep disorders are a severity marker in patients who use drugs and have an associated psychopathological disorder. It is necessary to continue researching the influence of insomnia in the severity of the psychopathology and relapses. Sleep disorders must be considered a “clinical marker” of the presence of a dual pathology in addict patients and, therefore, it is necessary to thoroughly assess the presence of other mental disorders in patients who consume drugs and report insomnia.

## Figures and Tables

**Table 1:** Descriptive representation of demographic, clinical, and psychiatric variables in each sleep disorder and in the total sample. N and percentages are represented for each variable. Abbreviations: IDDDU: Inpatient Dual Diagnoses Detoxification Unit; ODC: Outpatient Drug Clinic.

Characteristics		Sample	Insomnia/ Any Sleep Disorder	Delayed Sleep Induction	Sleep Fragmentation	Early Awakening	Nightmares
		N = 398	N = 218	N = 150	N = 137	N = 85	N = 80
Sex	Men	306 (76.9%)	175 (80.3%)	121 (80.7%)	107 (78.1%)	65 (76.5%)	53 (66.2%)
	Women	92 (23.1%)	43 (19.7%)	29 (19.3%)	30 (21.9%)	20 (23.5%)	27 (33.8%)
Age 18–27 years		22 (5.5%)	11 (5.0%)	9 (6.0%)	7 (5.1%)	4 (4.7%)	4 (5.0%)
28–37 years		60 (15.0%)	32 (14.7%)	23 (15.4%)	22 (16.1%)	8 (9.4%)	12 (15.0%)
38–47 years		128 (32.2%)	67 (30.7%)	45 (30.0%)	44 (32.1%)	28 (32.9%)	28 (35.0%)
48–57 years		138 (34.7%)	78 (35.8%)	57 (38.0%)	51 (37.2%)	37 (43.5%)	32 (40.0%)
58–67 years		39 (9.8%)	24 (11.0%)	14 (9.3%)	10 (7.3%)	7 (8.3%)	4 (5.0%)
≥68 years		11 (2.8%)	6 (2.8%)	2 (1.3%)	3 (2.2%)	1 (1.2%)	0 (0.0%)
Origin	IDDDU	198 (49.7%)	100 (45.9%)	58 (38.7%)	58 (42.3%)	32 (37.6%)	31 (38.8%)
	ODC	200 (50.3%)	118 (54.1%)	92 (61.3%)	79 (57.7%)	53 (62.4%)	49 (61.3%)
Dual Disorder		245 (61.6%)	146 (67.0%)	102 (68%)	96 (70.1%)	64 (75.3%)	64 (80%)
Affective disorder		154 (40.8%)	91 (42.7%)	65 (43.3%)	60 (44.1%)	42 (50.6%)	41 (51.9%)
Psychotic disorder		48 (12.7%)	26 (12.2%)	20 (13.3%)	19 (14.0%)	10 (12%)	13 (16.5%)
Personality disorder		86 (22.3%)	53 (24.3%)	40 (26.7%)	39 (28.5%)	24 (28.2%)	30 (37.5%)
Previous treatment		172 (43.2%)	103 (49.5%)	81 (55.9%)	69 (53.1%)	49 (61.3%)	48 (62.3%)
Multiple drug-users		193 (48.5%)	117 (53.7%)	88 (58.7%)	78 (57.0%)	49 (57.6%)	50 (62.5%)
Occupational status							
Working		75 (18.8%)	31 (14.2%)	22 (14.7%)	18 (13.1%)	10 (11.8%)	6 (7.5%)
Unemployed		151 (37.9%)	96 (44.0%)	68 (45.3%)	66 (48.2%)	45 (52.9%)	46 (57.5%)
On leave		43 (10.8%)	19 (8.7%)	11 (7.3%)	11 (8.0%)	5 (5.9%)	6 (7.5%)
Retired		108 (27.1%)	61 (28.0%)	40 (26.7%)	24 (17.5%)	19 (22.3%)	20 (25.0%)
Other conditions		21 (5.4%)	11 (5.1%)	9 (6.0%)	18 (13.1%)	6 (7.1%)	2 (2.5%)
Pharmacological treatment on first interview							
Alprazolam		17 (4.3%)	9 (4.1%)	7 (4.7%)	8 (5.8%)	4 (4.7%)	2 (2.5%)
Disulfiram		13 (3.3%)	3 (1.4%)	2 (1.3%)	2 (1.5%)	1 (1.2%)	2 (3.4%)
Clorazepate		7 (1.8%)	3 (1.4%)	2 (1.3%)	0 (0.0%)	0 (0.0%)	1 (1.3%)
Diazepam		13 (3.3%)	3 (1.4%)	1 (0.7%)	1 (0.7%)	0 (0.0%)	1 (1.3%)
Lorazepam		24 (6.0%)	15 (6.9%)	10 (6.7%)	8 (5.8%)	8 (9.4%)	6 (7.5%)
Lormetazepam		1 (0.3%)	5 (2.3%)	4 (2.7%)	2 (1.5%)	3 (3.5%)	2 (2.5%)
Methadone		2 (0.5%)	1 (0.6%)	1 (0.7%)	2 (1.5%)	0 (0.0%)	0 (0.0%)
Mirtazapine		8 (2.0%)	6 (2.8%)	6 (4.0%)	3 (2.2%)	3 (3.5%)	3 (3.8%)
Olanzapine		9 (2.3%)	7 (3.2%)	4 (2.7%)	5 (3.6%)	2 (2.4%)	3 (3.8%)
Pregabalin		4 (1.0%)	4 (1.8%)	3 (2.0%)	3 (2.2%)	0 (0.0%)	2 (2.5%)
Quetiapine		12 (3.0%)	4 (1.8%)	2 (1.3%)	1 (0.7%)	2 (2.4%)	2 (2.5%)
Tiapride		12 (3.0%)	7 (3.2%)	5 (3.3%)	4 (2.9%)	4 (4.7%)	4 (5.0%)
Trazodone		10 (2.5%)	7 (3.2%)	5 (3.3%)	5 (3.6%)	4 (4.7%)	3 (3.8%)

**Table 2:** Use and quantity of cannabis, cocaine, alcohol, opioids, and benzodiazepines consumed. Data for total sample and subgroups associated with sleep disorders appear in different columns. For alcohol and cannabis users there is an extra variable related with withdrawal. Abbreviations: CWA-Ar ≥ 10; Revised Clinical Institute Withdrawal Assessment for Alcohol Scale, upper 10 score.

Type of Consumers	Sample N = 398	Insomnia/any Sleep Disorder N = 218	Delayed Sleep Induction N = 150	Sleep Fragmentation N = 137	Early Awakening N = 85	Nightmares N = 80
	N (%)	N (%)	N (%)	N (%)	N (%)	N (%)
Cannabis Consumers	115 (28.9%)	65 (29.8%)	50 (33.3%)	43 (31.4%)	23 (27.0%)	25 (31.2%)
Quantity of Cannabis						
1–5 U	95 (23.9%)	55 (25.2%)	42 (28.0%)	36 (26.3%)	20 (23.5%)	22 (27.5%)
6–10 U	2 (0.5%)	1 (0.5%)	0 (0.0%)	0 (0.0%)	0 (0.0%)	0 (0.0%)
11–15 U	0 (0.0%)	0 (0.0%)	0 (0.0%)	0 (0.0%)	0 (0.0%)	0 (0.0%)
16–30 U	0 (0.0%)	0 (0.0%)	0 (0.0%)	0 (0.0%)	0 (0.0%)	0 (0.0%)
31 U or more	18 (4.5%)	9 (4.1%)	8 (5.3%)	7 (5.1%)	3 (3.5%)	3 (3.8%)
Cannabis withdrawal	38 (9.9%)	22 (10.1%)	17 (11.3%)	18 (13.1%)	7 (8.2%)	9 (11.3%)
Cocaine consumers	113 (28.4%)	83 (38.1%)	67 (44.7%)	49 (64.2%)	30 (35.3%)	36 (45.0%)
Quantity of Cocaine						
1 g/week	30 (7.5%)	19 (9.5%)	13 (9.6%)	13 (10.1%)	6 (7.6%)	9 (12.3%)
2 g/week	21 (5.3%)	13 (6.5%)	11 (8.1%)	9 (7.0%)	3 (3.8%)	3 (4.1%)
3 g/week	9 (2.3%)	7 (3.5%)	6 (4.4%)	4 (3.1%)	4 (5.1%)	4 (5.5%)
4 g/week	11 (2.8%)	6 (3.0%)	5 (1.5%)	1 (0.8%)	1 (1.3%)	3 (5.5%)
5 g/week	7 (1.0%)	4 (2.0%)	2 (1.5%)	4 (3.1%)	3 (3.8%)	1 (1.4%)
6 g/week or more	35 (8.8%)	17 (8.5%)	15 (11.1%)	10 (7.8%)	7 (8.9%)	9 (12.3%)
Alcohol consumers	236 (66.1%)	142 (65.1%)	93 (62.0%)	87 (63.5%)	57 (67.1%)	47 (58.7%)
Quantity of Alcohol						
1–20 units/day	220 (55.3%)	117 (53.7%)	77(53.1%)	71 (51.8%)	47 (55.3%)	38 (47.5%)
21–40 units/day	36 (9.0%)	21 (9.6%)	14 (9.3%)	14 (10.2%)	8 (9.4%)	8 (10.0%)
41 units/day or more	7 (1.8%)	3 (1.4%)	2 (1.3%)	2 (1.5%)	2 (2.4%)	1 (1.3%)
CIWA–Ar ≥ 10	169 (44.1%)	94 (43.1%)	58 (38.7%)	55 (40.1%)	30 (35.3%)	25 (31.3%)
Opioids consumers	72 (18.1%)	48 (22.0%)	40 (26.7%)	32 (23.4%)	21 (24.7%)	17 (21.2%)
Quantity of Opioids						
1–10 g/week	72 (18.1%)	48 (22.0%)	40 (26.7%)	32 (23.4%)	21 (24.7%)	17 (21.2%)
Benzodiazepines consumers	93 (23.7%)	61 (28.0%)	48 (32.0%)	46 (33.6%)	30 (35.3%)	31 (38.7%)
1–10 mg/day	47 (11.8%)	32 (14.7%)	24 (16.0%)	23 (16.8%)	15 (17.6%)	15 (12.4%)
11–21 mg/day	12 (3.0%)	8 (3.7%)	7 (4.7%)	8 (5.8%)	5 (5.9%)	4 (5.0%)
22–31 mg/day	1 (0.3%)	1 (0.5%)	1 (0.7%)	1 (0.7%)	1 (1.2%)	0 (0.0%)
32 mg or more	33 (8.3%)	20 (9.2%)	16 (10.7%)	14 (10.2%)	9 (10.6%)	12 (15.0%)

**Table 3:** Relationships between variables analyzed with non-parametric test chi-square and *p*-values. Abbreviations; IDDDU: Inpatient Dual Diagnoses Detoxification Unit; ODC: Outpatient Drug Clinic. *: significant values with *p* < 0.05.

Characteristics	Insomnia/Any Sleep Disorder (*n* = 218)		Delayed Sleep Induction (*n* = 150)		Sleep Fragmentation (*n* = 216)		Early Awakening (*n* = 85)		Nightmares (*n* = 80)	
	*χ* ^2^	*p*	*χ* ^2^	*p*	*χ* ^2^	*p*	*χ* ^2^	*p*	*χ* ^2^	*p*
Sex	3.023	0.082	1.781	0.182	0.121	0.728	0.016	0.898	7.069	0.008 *
Age	2.585	0.764	2.758	0.737	1.322	0.93	5.313	0.379	5.346	0.375
IDDDU/ODC	2.092	0.148	10.48	0.010 *	3.456	0.063	5.598	0.018 *	3.898	0.048 *
Dual Disorder	4.267	0.039 *	2.877	0.090	4.862	0.027 *	7.554	0.006 *	12.988	0.000 *
Previous treatment	2.442	0.118	9.291	0.002 *	4.180	0.041 *	9.913	0.002 *	10.814	0.001 *
Affective disorder	1.935	0.380	1.365	0.505	1.579	0.454	4.640	0.098	5.598	0.061
Psychotic disorder	0.122	0.727	0.056	0.813	247	0.619	0.033	0.855	1.353	0.254
Personality disorder	1.592	0.207	1.84	0.175	4.475	0.034 *	2.183	0.140	13.596	0.000 *
Multiple drug- users	4.254	0.039 *	9.368	0.002 *	5.469	0.019 *	3.532	0.060	7.603	0.006 *
Unemployed	13.557	0.019 *	8.867	0.14 *	12.438	0.029 *	12.823	0.025 *	25.732	0.000 *
Pharmacological treatment on first interview	29.55	0.009 *	24.06	0.064	28.737	0.017 *	19.306	0.2	13.295	0.579
Amount of cannabis consumed	16.935	0.390	22.760	0.120	11.728	0.762	7.919	0.951	19.666	0.236
Cannabis withdrawal	0.016	0.898	0.490	0.484	2.345	0.126	0.401	0.527	0.255	0.614
Amount of alcohol consumed	1.447	0.695	2.018	0.569	1.491	0.684	0.589	0.899	2.827	0.419
CIWA-Ar ≥ 10	0.208	0.649	2.805	0.094	1.257	0.262	3.511	0.061	6.488	0.011 *
Amount of cocaine consumed	3.852	0.697	9.53	0.16	7.294	0.294	5.99	0.424	8.41	0.21
Amount of opioids consumed	0.122	0.727	0.056	0.813	0.247	0.619	0.33	0.855	1.353	0.245
Amount of benzodiazepines consumed	9.848	0.043 *	15.21	0.04 *	16.924	0.002 *	13.316	0.010 *	18.980	0.001 *

**Table 4:** Several binomial logistic regression analyses with the dependent variable varying among sleep disorder subgroups. Abbreviations; IDDDU: Inpatient Dual Diagnoses Detoxification Unit; ODC: Outpatient Drug Clinic.

Dependent Variable	Wald	*p*	OR	IC 95%
**Insomnia/any sleep disorder**				
Benzodiazepine use disorder	4.779	0.029	0.354	0.140–0.898
Dual Disorder	4.246	0.039	1.553	1.022–2.361
Treatment with trazodone	4.626	0.031	0.129	0.020–0.834
Treatment with pregabalin	4.626	0.031	0.129	0.020–0.834
**Delayed sleep induction**				
Origin: ODC/IDDDU	10.364	0.001	1.991	1.309–3.027
Previous treatment	9.198	0.002	0.519	0.34–0.793
Benzodiazepine use disorder	7.189	0.007	0.319	0.138–0.735
Treatment with pregabalin	3.857	0.05	0.091	0.008–0.995
**Sleep fragmentation**				
Dual Disorder	4.824	0.028	0.606	0.388–0.948
Previous treatment	4.158	0.041	0.639	0.515–0.983
Benzodiazepine use disorder	4.567	0.033	0.423	0.192–0.931
Treatment with pregabalin	3.857	0.05	0.091	0.008–0.995
Personality disorder	4.426	0.035	0.590	0.361–0.965
**Early awakening**				
Dual Disorder	7.353	0.007	0.470	0.273–0.811
Origin: ODC/IDDDU	5.519	0.019	1.810	1.103–2.970
Previous treatment	9.657	0.002	0.447	0.269–0.743
Benzodiazepine use disorder	3.900	0.048	0.433	0.189–0.994
**Nightmares**				
Origin: ODC/IDDDU	3.856	0.050	1.656	1.001–2.741
Previous treatment	10.490	0.001	0.425	0.254–0.714
Dual Disorder	12.256	0.000	0.346	0.191–0.627
Benzodiazepine use disorder	9.455	0.002	0.278	0.123–0.629
Personality disorder	4.426	0.035	0.590	0.361–0.965
